# Silver nanoparticles of different sizes induce a mixed type of programmed cell death in human pancreatic ductal adenocarcinoma

**DOI:** 10.18632/oncotarget.22563

**Published:** 2017-11-20

**Authors:** Ewelina Zielinska, Agata Zauszkiewicz-Pawlak, Michal Wojcik, Iwona Inkielewicz-Stepniak

**Affiliations:** ^1^ Department of Medical Chemistry, Medical University of Gdansk, Debinki 1, 80-211 Gdansk, Poland; ^2^ Department of Histology, Medical University of Gdansk, Debinki 1, 80-211 Gdansk, Poland; ^3^ Faculty of Chemistry, University of Warsaw, Pasteura 1, 02-093 Warsaw, Poland

**Keywords:** pancreas ductal adenocarcinoma cells, silver nanoparticles, autophagy, necroptosis, mitotic catastrophe

## Abstract

Pancreatic ductal adenocarcinoma, with the high resistance to chemotherapeutic agents, remains the fourth leading cause of cancer-death in the world. Due to the wide range of biological activity and unique properties, silver nanoparticles (AgNPs) are indicated as agents with potential to overcome barriers involved in chemotherapy failure. Therefore, in our study we decided to assess the ability of AgNPs to kill pancreatic cancer cells, and then to identify the molecular mechanism underlying this effect. Moreover, we evaluated the cytotoxicity of AgNPs against non-tumor cell of the same tissue (hTERT-HPNE cells) for comparison. Our results indicated that AgNPs with size of 2.6 and 18 nm decreased viability, proliferation and caused death of pancreatic cancer cells in a size- and concentration-dependent manner. Ultrastructural analysis identified that cellular uptake of AgNPs resulted in apoptosis, autophagy, necroptosis and mitotic catastrophe. These alterations were associated with increased pro-apoptotic protein Bax and decreased level of anti-apoptotic protein Bcl-2. Moreover, AgNPs significantly elevated the level of tumor suppressor p53 protein as well as necroptosis- and autophagy-related proteins: RIP-1, RIP-3, MLKL and LC3-II, respectively.

In addition, we found that PANC-1 cells were more vulnerable to AgNPs-induced cytotoxicity compared to pancreatic non-tumor cells.

In conclusion, AgNPs by inducing mixed type of programmed cell death in PANC-1 cells, could provide a new therapeutic strategy to overcome chemoresistance in one of the deadliest human cancer.

## INTRODUCTION

Despite the huge progress that has been made in recent decades in the diagnosis, treatment and prevention of cancer, the survival rate for pancreatic cancer is still very low. According to the statistics, it is the fourth leading cause of cancer deaths in the world, and about 3% of all malignant tumors occurring in humans [1]. Unfortunately, it has been observed an increase of morbidity and mortality from pancreatic cancer [2]. The most common and deadly form of pancreatic cancer is pancreatic ductal adenocarcinoma occurs in approximately 80-90% of cases [3]. Surgical resection of tumor provides the only chance for cure, regretfully, possible in about 15-20% of patients. Moreover, pancreatic cancer responds poorly to most chemotherapeutic agents. There are different mechanisms adapted by cancerous cells to resist treatment, including alteration in drug transport and an excess of anti-apoptotic proteins and/or deficient in pro-apoptotic proteins, for example Bax/Bcl-2 protein [3, 4]. Anticancer drug to be effective it must be taken up by all cells in a tumor and has to trigger of cellular pathways leading to death. Thus, apart from apoptosis other types of programmed cell death, particularly necroptosis has received increased attention as targets for anti-cancer therapy. Today we know that necrosis can be induced in a controlled manner by specific genes and regulated by kinases RIP-1 and RIP-3 and MLKL protein [5]. This process has been designated as programmed necrosis (III type) or necroptosis, or regulated necrosis and represents a therapeutic alternative to apoptosis-resistant forms of cancer [5, 6]. Furthermore, autophagy is another form of programmed cell death (II type), which has been observed in pancreatic cancer cells during treatment with chemotherapeutic agents [7]. Some studies indicated that autophagy initiation is detrimental to pancreatic ductal adenocarcinoma and increase the efficacy of anticancer drugs [8–10]. On the other hand, it has been demonstrated that autophagy facilitated pancreatic cancer cell survival and enhanced their chemoresistance [11]. Thus, the role of autophagy in treatment-induced pancreatic cancer cell death is very complex ant still not clear. Although, all the three processes of programmed cell death are distinct, their co-existence and cross-talk is also observed [12–16]. Moreover, mitotic catastrophe in response to treatment with anticancer drug was found in pancreatic tumor cell [17]. It is distinct from apoptosis, autophagy, necroptosis and necrosis, oncosuppressive mechanism, which restrains tumorigenesis and cancer progression [18]. Inducers of mitotic catastrophe opening new strategies and perspective for anticancer therapy and there are several that being evaluated in preclinical and clinical studies [19]. To conclude, the knowledge of molecular mechanism regarding cancer cell death is crucial for the development and progress in pancreatic anticancer therapy [20].

With the development of nanotechnology, silver nanoparticles (AgNPs) have gained a growing interest as a promising agents for anticancer therapy [21, 22]. NPs are unique because of their high surface area to volume ratio (small size) and ability to easily penetrate the cell membranes and the biological barriers. This properties increase effectiveness against the tumor cells at lower concentration and subsequently, reduced toxicity to surrounding non-cancer cells. It has been presented that AgNPs showed a wide spectrum of biological activities and a strong inhibitory effect on the growth of human lung cancer (H1299), human tongue squamous carcinoma (SCC-25), prostate cancer (VCaP), breast cancer (MCF-7)[23–25]. However, the molecular mechanisms of cancer cell death caused by treatment with AgNPs remain not fully understood and known.

Considering the information mentioned above, we decided to investigate the influence of AgNPs on human pancreatic ductal adenocarcinoma cell death, including apoptosis, necroptosis, autophagy and mitotic catastrophe. Moreover, we evaluated also the cytotoxicity of AgNPs against non-tumor cell of the same tissue (hTERT cell line).

## RESULTS

### Characterization of AgNPs

Characterization of 18 nm AgNPs is shown in our previous work [27]. Additionally, physicochemical properties of 18 nm AgNPs under experimental conditions of the present study are summarized in Table 1.

**Table 1:** Physicochemical characterization of AgNPs

Characterization		Results	
		PANC-1	hTERT-HPNE
**Polydispersity index (PDI)**		0.211±0.021	0.223±0.057
**Zeta potential (mV)**		-31.1±1.0	-28.9±3.4
^*^ Soluble Ag released (%)			
2 nm (5 μg/mL); 24 h		2.8	2.8
15 nm (50 μg/mL); 24 h		0.66	0.71
**Hydrodynamic diameter of 18 nm AgNPs (nm)**			
10 μg/mL	· „0” h:	22±4	24±6
	· 24 h:	49±6	38±9
50 μg/mL	· „0” h:	26±8	33±4
	· 24 h:	66±9	71±8
100 μg/mL	· „0” h:	29±9	38±7
	· 24 h:	84±6	78±9

Note: Data are presented as mean ±SD; (n=5). The zeta potential, the PDI and the hydrodynamic diameter were measured after dispersion of 18 nm AgNPs at concentration of 50 μl/mL or as indicated in serum free cell culture medium using Zetasizer. ^*^The amount of released Ag in SF cell medium after 24 h; 37°C, 5% CO2 and centrifugation was quantified by ICP-MS and expressed as percentage from AgNPs, n=4.a) Note: Data are presented as mean ±SD; (n=5). The zeta potential, the PDI and the hydrodynamic diameter were measured after dispersion of 18 nm AgNPs at concentration of 50 μl/mL or as indicated in serum free cell culture medium using Zetasizer. ^*^The amount of released Ag in SF cell medium after 24 h; 37°C, 5% CO2 and centrifugation was quantified by ICP-MS and expressed as percentage from AgNPs, n=4.

**Figure 1:**
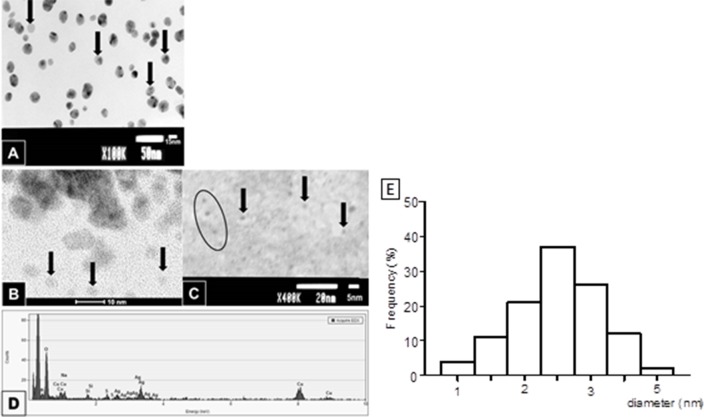
Characterization of AgNPs by TEM AgNPs of 18 nm in diameter, the arrows point at a single nanoparticles some AgNPs may aggregate forming clumps of two or three, thus giving the impression of the greater size **(A)**. 2.6 nm AgNPs are hardly visible due to the low contrast of the sample, enabling better resolution on the other hand **(B** and **C**; arrows and ellipse). EDS analysis of AgNPs confirmed the presence of silver elements (Ag) in the examined area. The remaining elements are derived from the milieu (i.e. evaporated water, formvar coated copper mesh and microscopic column) **(D)**. The histogram shows that the size for smaller AgNPs ranged from 1-5 nm with an average particle size of 2.6 nm **(E)**. Size distributions were obtained from TEM micrograph.

The zeta potential and the PDI value indicated that 18 nm AgNPs are stable and monodispersed in SF culture medium, the hydrodynamic size of AgNPs increased in concentration- and time-dependent manner and this results are similar to our previous finding and conclusion [27]. The content of soluble Ag in culture medium after 24 h incubation with 2.6 and 18 nm AgNPs was detected to be 2.8% and 0.66 - 0.71%, respectively and did not affect the cytotoxicity of AgNPs (Supplementary Figure 1 and 2). During TEM equipped with EDS analysis we confirmed spherical shape and the presence of silver elements (Ag) (Figure 1A-1D). We found a diameter range of 1 - 5 nm with a mean size of 2.6±0.8 nm for smaller AgNPs (Figure 1E), and a diameter range of 10 - 26 nm with a mean diameter of 18±2.6 nm for the bigger one as described in our previous study [27].

Moreover, we noticed that the physiochemical properties of AgNPs in cell culture medium for PAN-1 and hTERT-HPNE cells were very similar (Table 1). We also confirmed that the presence of necrostatin-1 (10 μM) had no significant impact on investigated physicochemical parameters (data not shown).

### AgNPs decreased viability of PANC-1 cells more significantly than hTERT cells

**Figure 2:**
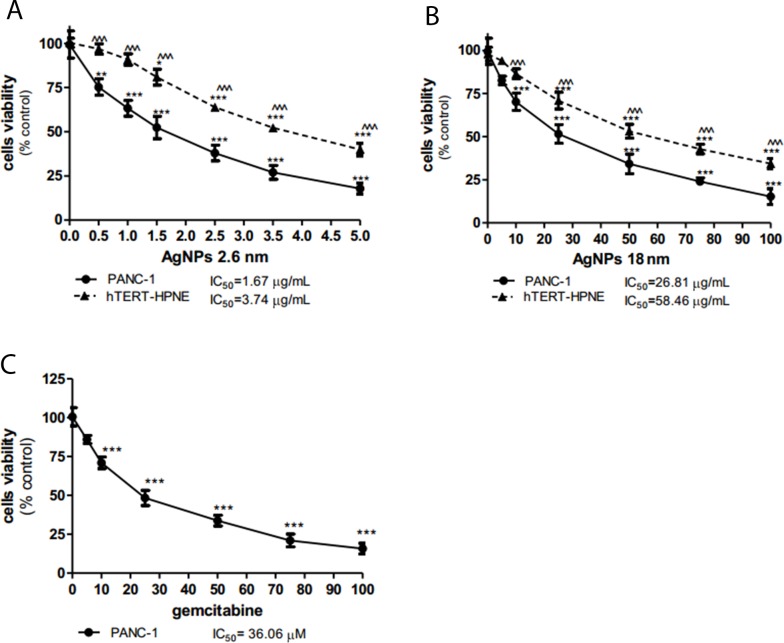
AgNPs decreased PANC-1 cells viability more significantly than hTERT-HPNE cells Concentration-dependent cytotoxicity of 2.6 nm AgNPs 18 nm AgNPs against PANC-1 **(A)** and hTERT-HPNE cells **(B)** and gemcitabine against PANC-1 cells **(C)** after 24 h of incubation. Data are expressed as means ± SD of 3 independent experiments. ^*^p<0.05; ^**^p<0.01; ^***^p<0.001 exposed cells v/s control. ^^^p<0.001 exposed PANC-1 cells v/s exposed hTERT-HPNE cells.

First, we evaluated the effects of 2.6 and 18 nm AgNPs on PANC-1 and hTERT-HPNE cells viability using MTT assay. We found that both 2.6 and 18 nm AgNPs decreased cell viability in a size- and concentration-dependent manner (Figure 2). AgNPs with size 2.6 nm exhibited stronger cytotoxicity against PANC-1 cells resulted in an IC_50_ of 1.67 μg/mL than 18 nm AgNPs with IC_50_ of 26.81 μg/mL after 24 h exposure. It has to be noted that non-tumor cells were more resistance to cytotoxic effect of 2.6 and 18 nm AgNPs with more than 2-fold higher IC value equaling 3.74 μg/mL and 58.46 μg/mL, respectively. 2.6 nm AgNPs exerted about 16-fold higher cytotoxicity than 18 nm AgNPs. To compare the cytotoxic effect of AgNPs and Ag^+^, we determined the viability of PANC-1 and hTERT-HPNE cells after exposure to AgNO_3_ (Supplementary Figure 3). The IC_50_ was 1.49 and 3.22 μg/mL for PANC-1 and hTERT-HPNE, respectively. Our results showed similar effect of 2.6 nm AgNPs and Ag ions. Furthermore, we indicated that Ag^+^ was about eighteen-fold more toxic than 18 nm AgNPs to both investigated pancreatic cell lines. In addition PANC-1 cell line was treated with gemcitabine (drug which is commonly used to treat pancreatic cancer) as a positive control (Figure 2C). It decreased cells viability in a concentration-dependent manner with the IC_50_ value equaling 36.06 μM (9400000 μg/mL). Concentration of gemcitabine used in chemotherapy decreased PANC-1 cell viability around 5 × 10^6^ fold weaker than 2.6 nm AgNPs and 3 × 10^5^ fold weaker than 15 nm AgNPs. These data are consistent with the literature reports indicating the IC_50_ values for gemcitabine in the range of 9.5 nM - 300 mM [38–41].

### PANC-1 cells are more sensitive to AgNPs-induced cell death than hTERT cells

**Figure 3:**
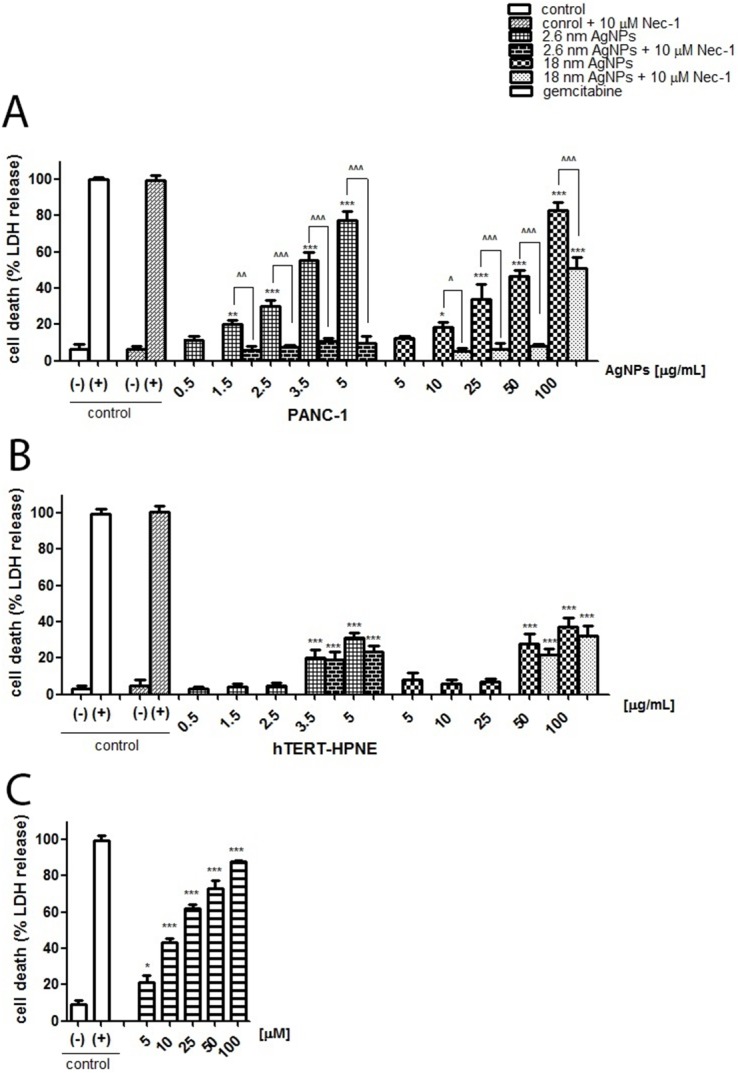
AgNPs induced PANC-1 cells death more significantly than hTERT-HPNE cells Necrostain-1 abrogated or protected against AgNPs-induced cells death in PANC-1 cells but not in hTERT cells. Concentration-dependent cytotoxicity, expressed as LDH release, of 2.6 nm AgNPs and 18 nm AgNPs against PANC-1 **(A)**, hTERT cells **(B)** and gemcitabine against PANC-1 cells **(C)** after 24h of incubation. Preincubation with necrostatin-1 (Nec-1) abrogated or attenuated AgNPs-induced cytotoxicity in PANC-1 **(A)** but not in hTERT cells **(B)**. Data are expressed as means ± SD of 3 independent experiments. ^*^p<0.05; ^**^p<0.01; ^***^p<0.001 exposed cells v/s untreated control (-) or ^p<0.05; ^^p<0.01; ^^^p<0.001 as indicated.

Subsequently, we have assessed the effect of AgNPs on PANC-1 and hTERT-HPNE cells death. We found that both 2.6 and 18 nm AgNPs induced cells death, measured by LDH release from cells into the culture medium, in a size- and concentration-dependent manner (Figure 3A and 3B). Compared to untreated control, a significant increase in PANC-1 cell death was observed following 1.5, 2.5, 3.5, 5 μg/mL 2.6 nm AgNPs treatment (about 15, 24, 49, 72 %, respectively) and 10, 25, 50, 100 μg/mL 18 nm AgNPs treatment (about 12, 28, 43, 77 %, respectively). Non-cancerous cells were more resistant to AgNPs-induced cytotoxicity and statistically significant elevation of cell death was found after treatment with 3.5 and 5 μg/mL of 2.6 nm AgNPs (about 20%) or 50, 100 μg/mL of 18 nm AgNPs (about 46.7, 21.36 % respectively). Also, gemcitabine caused cells death in a concentration-dependent manner about 21, 46, 62, 73, 88 % after treatment with 5, 10, 25, 50, 100 μM, respectively (Figure 3C). This is a concentration of about 10^5^ fold higher than when using 18 nm AgNPs and of about 10^6^ fold higher when we used 2.6 nm AgNPs.

### AgNPs are more cytotoxic against PANC-1 cells than hTERT cells

In summary, the experiments described above together with IC_50_ values obtained for AgNPs from cell viability (MTT) and from cell death (LDH) assays using GraphPad program, indicated their higher cytotoxic effect on human pancreatic carcinoma cells than on non-tumor cell line of the same tissue (Table 2). Therefore, we have become interested in studying more deeply the cytotoxic potential of AgNPs towards PANC-1 cells.

**Table 2:** IC50 values obtained after exposure of PANC-1 and hTERT-HPNE cells to AgNPs

Cell line	AgNPs size (nm)	IC_50_ from LDH (μg/mL)	IC_50_ from MTT (μg/mL)
PANC-1	2.6	3.19	1.67
	18	56.46	26.81
hTERT-HPNE	2.6	8.06	3.74
	18	160.3	58.46

The inhibitory concentration, IC50, was calculated from the following equation: log(inhibitor) vs responses curve using the GraphPad Prism 5 program.

### AgNPs inhibited PANC-1 cell proliferation

**Figure 4:**
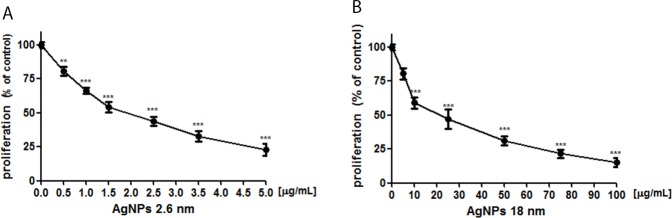
AgNPs reduced PANC-1 cells proliferation Incubation of PANC-1 cells with 2.6 nm AgNPs **(A)** or 18 nm AgNPs **(B)** for 24 h resulted in a concentration-dependent reduction of cells proliferation. Data are presented as mean ± standard deviation of 3 independent experiment. ^**^p<0.01; ^***^p<0.001 exposed cells v/s control.

Next, we measured BrdU incorporation test in order to elucidated antiproliferative activity of 2.6 nm and 18 nm AgNPs in PANC-1 cells. We have noted that exposure of PANC-1 cells to both 2.6 and 18 nm AgNPs for 24 h significantly decreased proliferation of these cells in a concentration- and size-dependent manner (Figure 4) with IC_50_ of 1.91 and 21.76 μg/mL, respectively.

### Ultrastructural alterations indicated AgNPs-induced mixed type of PANC-1 cell death

**Figure 5:**
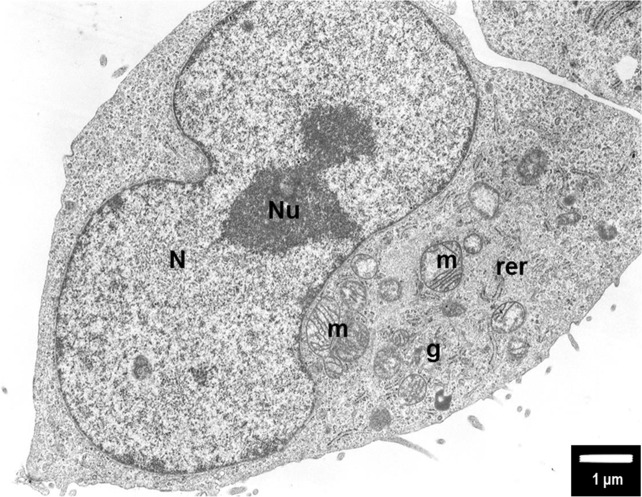
The untreated (control) PANC-1 cells shows normal morphology with euchromatic nucleus (N) and nucleolus (Nu), round mitochondria (m) with well-organized cristae and electron-lucent matrix Rough endoplasmic reticulum (rer) is dispersed within the cytoplasm together with Golgi apparatus (g). Magnification ×6000.

**Figure 6:**
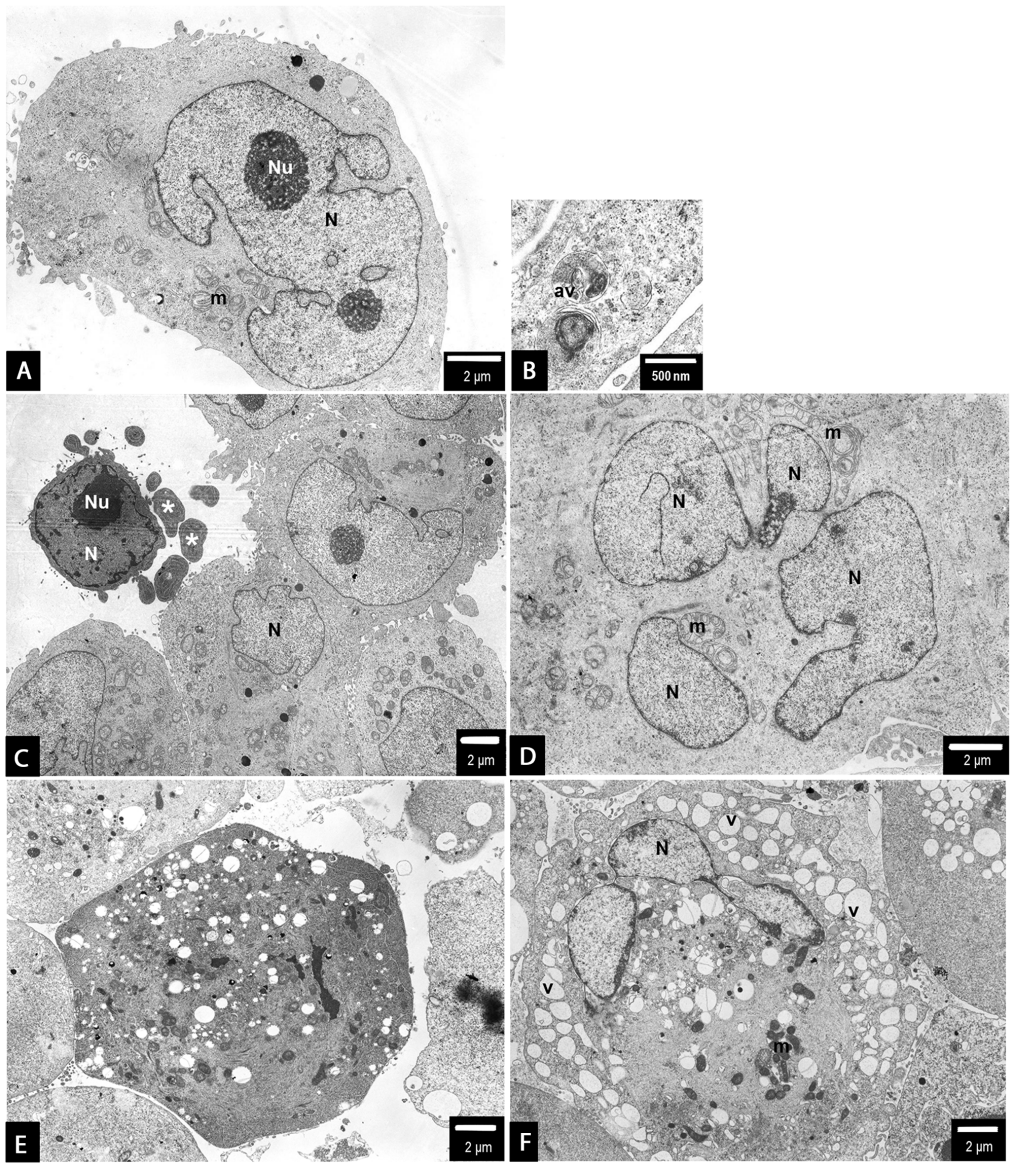
PANC-1 cells treated with 0.5 μg/mL **(A-D)** or 1.5 μg/mL **(E, F)** 2.6 nm AgNPs for 24 h. In general, no major changes have been observed in the overall cell morphology (a-c). However, some apoptotic cells were spotted occasionally, showing the characteristic condensation c-e of the nucleus (N) and cytoplasm and blebbing (c-e). Moreover, some autophagic vacuoles have been observed (b) as well as an evidence of mitotic catastrophe (increased size of the cell and multinucleation - N) accompanied by swirling of mitochondrial (m) cristae (d). Besides apoptotic cell, after treatment with 1.5 μg/mL, an evidence of non-apoptotic cell death: peripheric location of the nucleus (N), intensive vacuolation (v) of the cytoplasm, mitochondrial (m) condensation, gain in the cell size, all these features may be considered as an example of necroptosis among PANC-1 cells **(F)**. Magnifications: a ×4000; b ×15000; c ×2000; d - f ×3000.

**Figure 7:**
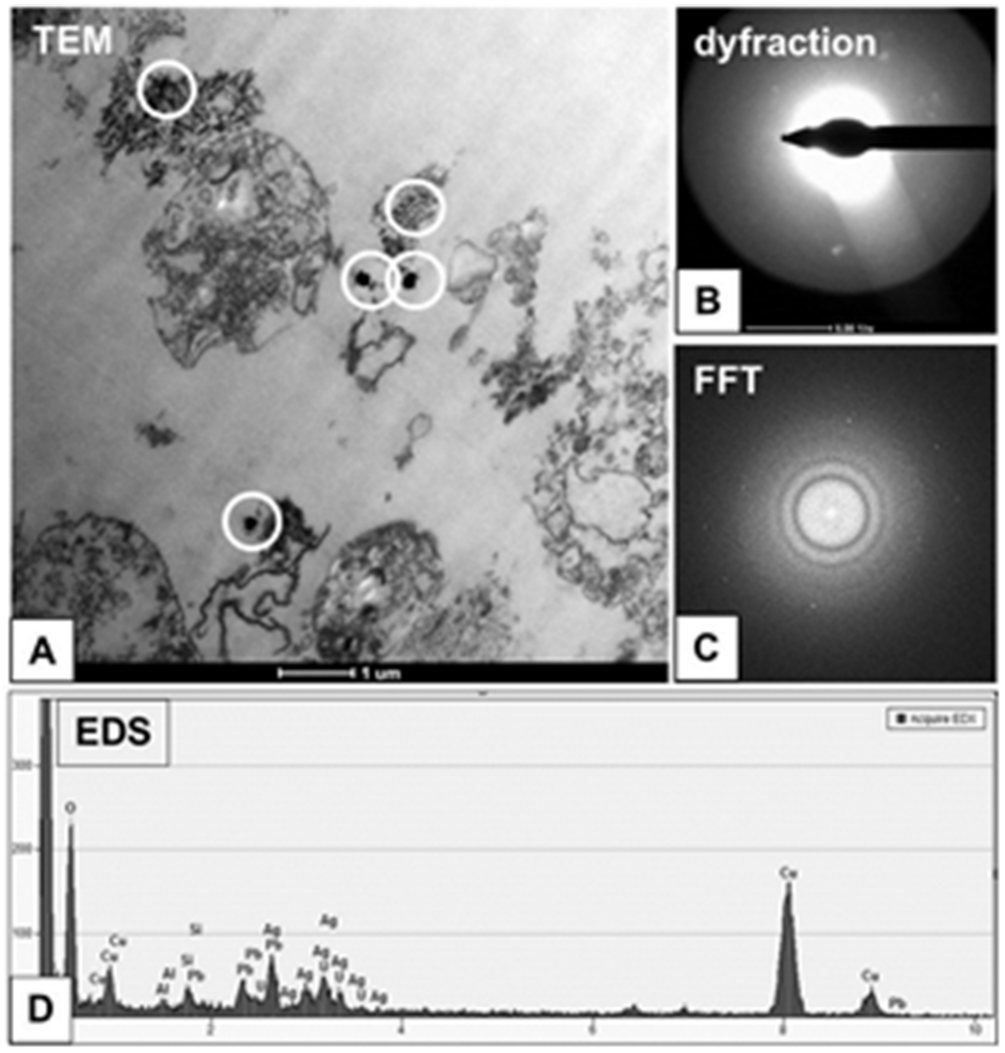
Characterization of 2.6 nm AgNPs in PANC-1 cells Aggregated AgNPs bound to the cellular membranes were tracked (circled) and examined **(A)**. The electron diffraction **(B)**, fast Fourier transformation (FFT) **(C)** and EDS analysis **(D)** of examined areas confirmed the presence of silver (Ag) elements. The remaining elements are derived from the milieu (i.e. formvar coated copper mesh, contrasting reagents and a microscopic column).

**Figure 8:**
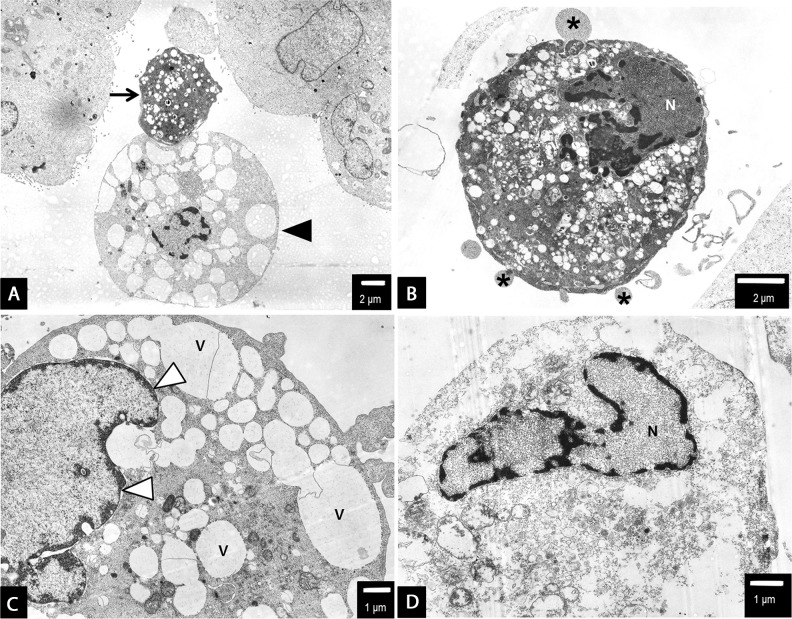
PANC-1 cells treated with 2.6 nm AgNPs AgNPs triggered both apoptotic and non-apoptotic cell death at 2.5 μg/mL **(A-C)** and 3.5 μg/mL **(D)** concentration. The evidence of both apoptotic (arrow) and necrotic/necroptotic (black arrow head) cell morphology have been found (a and b). Apoptotic cell with typical nuclear condensation (N), blebbing (asterisk) (b). The necroptotic cell death was preceded by intensive cytoplasm vacuolization (v), nuclear membrane dilatation (white arrow heads) and disrupted cellular membrane ad cytoplasmic swelling (a, c and d). Final stage of cellular death, degradation of cell organelles and cytoplasm (d). Loss of cell membrane integrity, chromatin condensation of nucleus (N). The representative cell is shown, presenting final stage of cellular death, degradation of cell organelles and cytoplasm, total loss of cell membrane integrity. However, the nuclear membrane integrity was maintained, the nucleus (N) was condensed, with peripheral clumps of heterochromatin. At this level of cell damage it was impossible to specify the cell death pathway. Magnifications: a ×2000; b ×4000; c ×5000; d ×6000.

**Figure 9:**
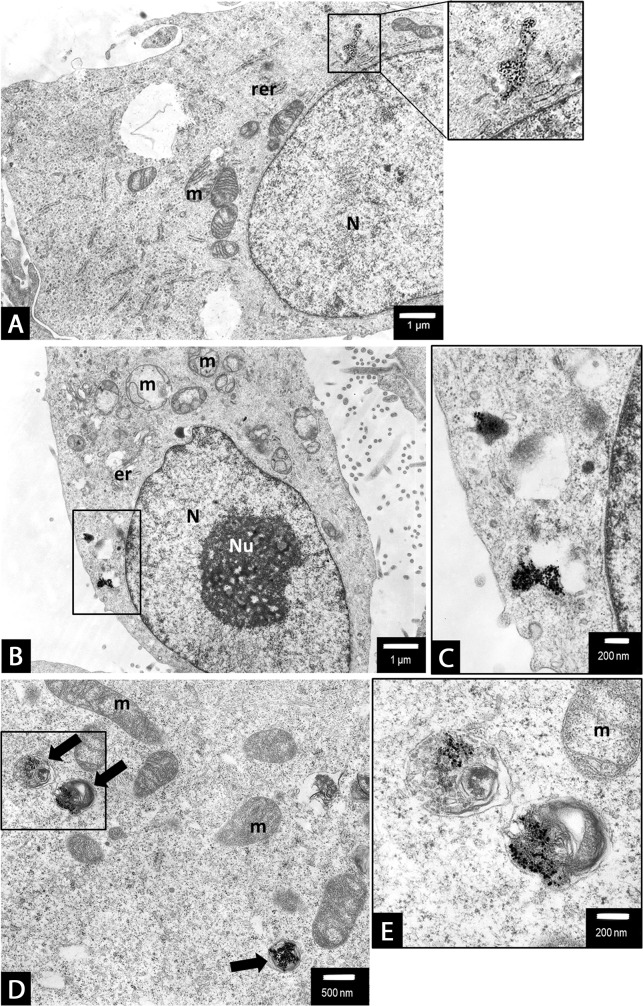
PANC-1 cells treated with of 18 nm AgNPs at 5 μg/mL concentrations Autophagosome formation. An evidence of 18 nm AgNPs clusters, encapsulated in a single membrane vacuoles of irregular shape (a-e and b, d show magnified boxed areas). Moreover, we observed clusters of AgNPs inside a double-membrane, multivesicular vacuoles, containing swirled membranes which reportedly are early autophagovacuoles (arrows) (d, e - shows magnified boxed area). Whereas other cell organelles such as mitochondria (m), endoplasmic reticulum (er, rer) remained intact **(A-E)**. Magnifications: a, b ×6000; c ×20000; d ×10000, e ×25000.

**Figure 10:**
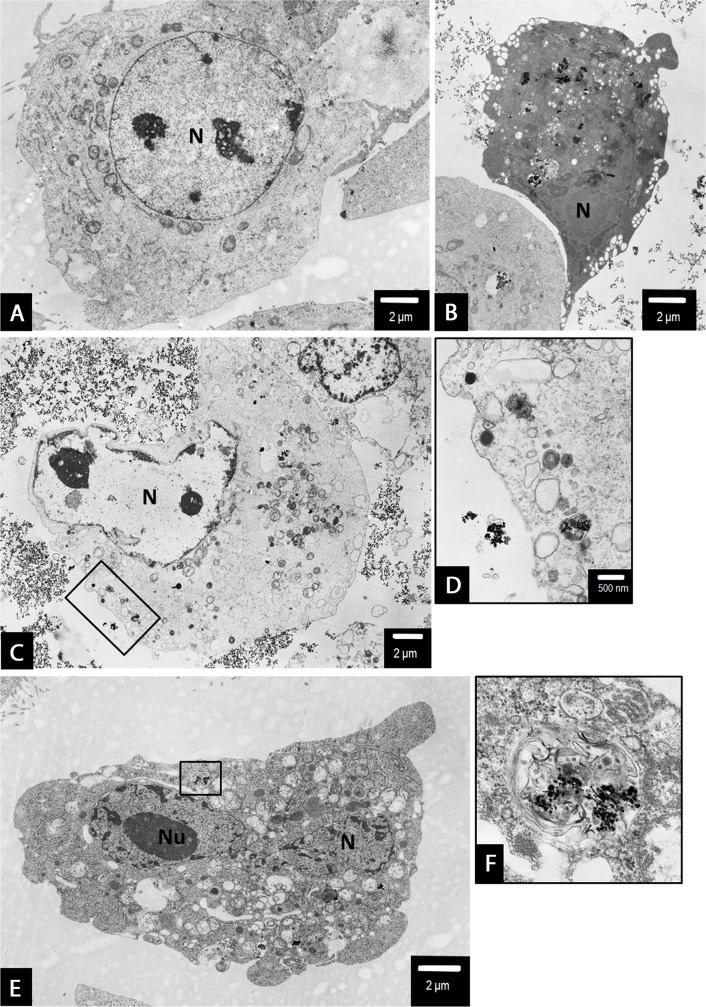
The evidence of PANC-1 cell death following treatment with 18 nm AgNPs at 15 μg/mL **(A, B)** and 25 μg/mL **(C-F)** concentrations. A non-apoptotic type of cell death have been observed with characteristic, electron-lucent cytoplasm and nuclei (N) with preserved integrity of nuclear membrane (a and c). An autophagosomes containing numerous AgNPs were present in the cytoplasm of dying cells (d, magnified boxed area from c). An apoptotic cell with numerous AgNPs aggregates (visible as black, contrast spots) present in shrunk, condensed cytoplasm and characteristic fragmented nucleus (N) has been observed (b and e). An autophagosome containing numerous AgNPs was present in the cytoplasm of apoptotic cell (f, magnified boxed area from e). Magnifications: a ×3000; b ×2500; c ×3000; d ×10000; e ×3000; f ×25000.

**Figure 11:**
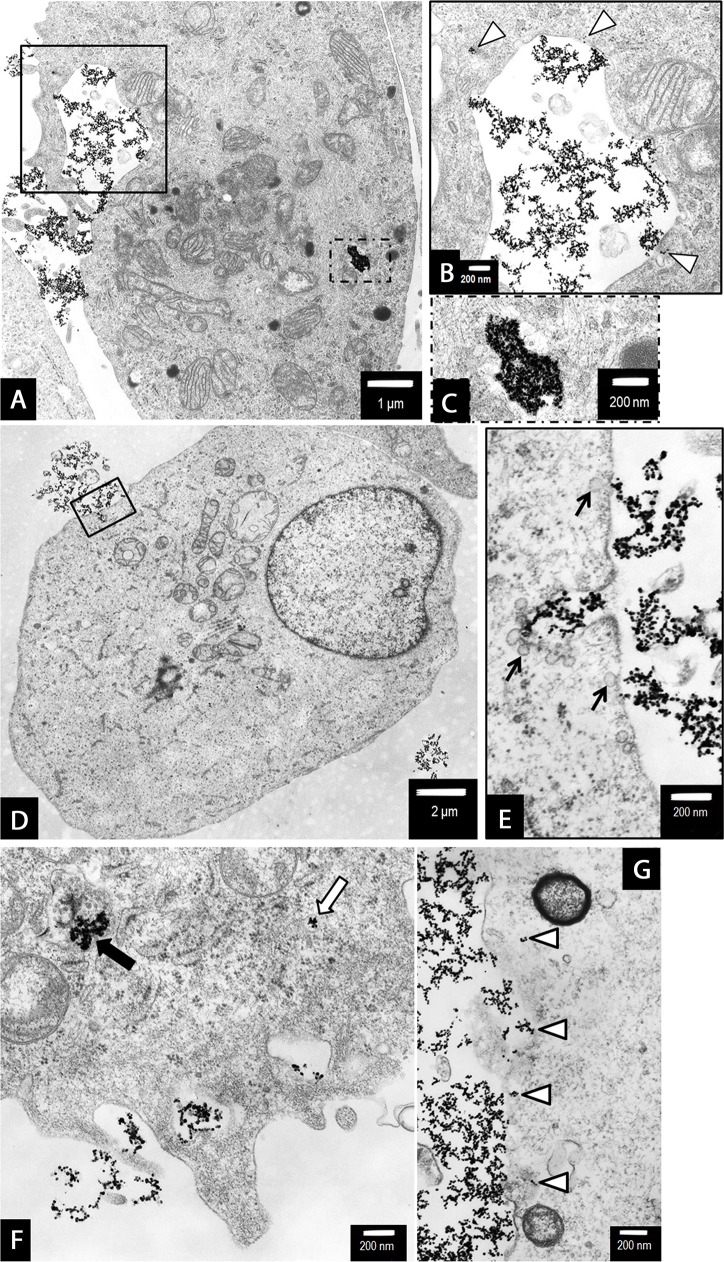
The uptake and internalization of 18 nm AgNPs in PANC-1 cells We suggest the sequence of events leading to the uptake of AgNPs was as follows: The AgNPs accumulated along the cell membrane **(A and G)**. Then AgNPs were internalized via caveoles (b, arrow heads and e, arrows) or penetrated freely through the cell membrane (g, arrow heads). Internalized AgNPs were then accumulated in the cytoplasm as single-membrane bounded vesicles (a, the dotted line box and c, magnified boxed area), free nanoparticles (arrow heads on b and g), small vesicles (f, white arrow) as well as within multivesicular vacuoles (atophagovacuoles) (f, black arrow). Magnifications: a ×6000; b, c ×20000; d ×3000; e-g ×25000.

To further characterize the effects of 2.6 and 18 nm AgNPs on PANC-1 cells death we performed ultrastructural analysis using TEM or TEM with the electron diffraction, fast Fourier transformation and EDS analysis. We found that NPs are uptake by PANC-1 cells (Figures 7, 9 and 11) and induced ultrastructural alterations depending on the concentration and the size when compared with control cells (Figures 5-10). For the bigger AgNPs we confirmed their internalization via caveoles (Figure 11B and 11E) or penetration freely through the cell membrane (Figure 11G) and localization in the cytoplasm as single-membrane bounded vesicles (Figure 8A, 8C), free nanoparticles (Figure 11B and 11G), small vesicles (Figure 11F) as well as within multivesicular vacuoles (Figure 11F). We observed features of apoptosis, necrosis and/or necroptosis associated with autophagasomes formation after treatment with either 2.6 or 18 nm AgNPs as shown on Figures 6-10. Moreover, we found PANC-1 cells increase in size with multiple nuclei (multinucleation) after exposure to 2.6 nm AgNPs at concentration of 0.5 μg/mL (Figure 6D). This result clearly indicated implication of mitotic catastrophe in AgNPs-induced cytotoxicity in pancreatic ductal adenocarcinoma cells.

### AgNPs induced apoptosis in PANC-1 cells

**Figure 12:**
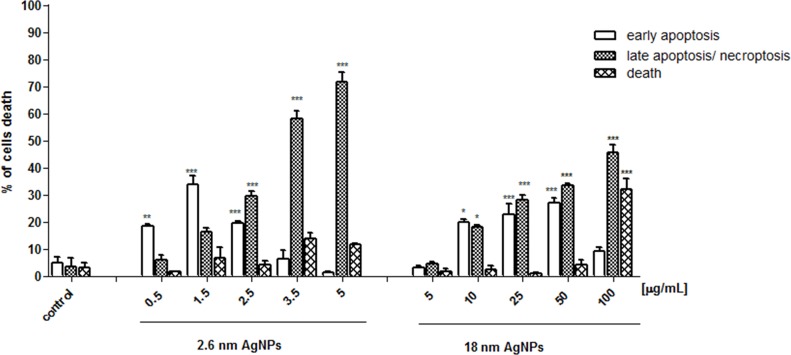
AgNPs-induced apoptosis in PANC-1 cells Data are expressed as means ± SD of 3 independent experiments. ^*^p<0.05; ^**^p<0.01; ^***^p<0.001 exposed cells v/s control.

Due to the ultrastructural changes characteristic for apoptosis, necroptosis/necrosis, we measured the amount of early apoptotic (Annexin V+ PI-), late apoptotic/necroptotic (Annexin V+ PI+) and dead (Annexin V- PI+) PANC-1 cells. As shown in Figure 12, the percentage of early apoptotic cells increased significantly after treatment with 2.6 nm AgNPs in the range of 0.5 - 2.5 μg/mL and the highest elevation we observed at concentration of 1.5 μg/mL. The percentage of cells undergoing late apoptosis and/or necroptosis increased in a concentration-dependent manner and prevailed over early apoptosis after exposure to 2.5, 3.5 and 5 μg/mL AgNPs. Moreover, we found that treatment with the bigger NPs in the range of 10 - 50 μg/mL resulted in a significant and very similar increase of both early apoptotic and late apoptotic/necroptotic cells. After exposure to 100 μg/mL AgNPs, the highest concentration used in our experiments, we observed elevation of late apoptotic/necroptotic cells and cells stained with PI (Annexin V- PI+).

### AgNPs induced necroptosis in PANC-1

Because ultrastructural features of necroptosis and necrosis are very similar, moreover, flow cytometry analysis indicated potential implication of necroptosis in AgNPs-induced PANC-1 cells death we measured LDH release into the culture medium in the presence of necrostatin-1 (Nec-1), as shown on Figure 3A. We found that preincubation with Nec-1 for 2 hours abrogated 2.6 nm AgNPs-induced PANC-1 cells death as well as cell death induced by larger ones at concentration until 50 μg/mL and significantly reduced cytotoxicity caused by the highest concentration - 100 μg/mL (about 62%). Importantly, we also noticed that preincubation with Nec-1 did not affect AgNPs-caused cell death in hTERT cells (Figure 3B). This results clearly indicated that AgNPs induced necroptosis in pancreatic cancer cells and necrosis in non-tumor cells of the same type of tissue.

### AgNPs induced changes in protein levels related to apoptosis, necroptosis and autophagy in PANC-1

**Figure 13:**
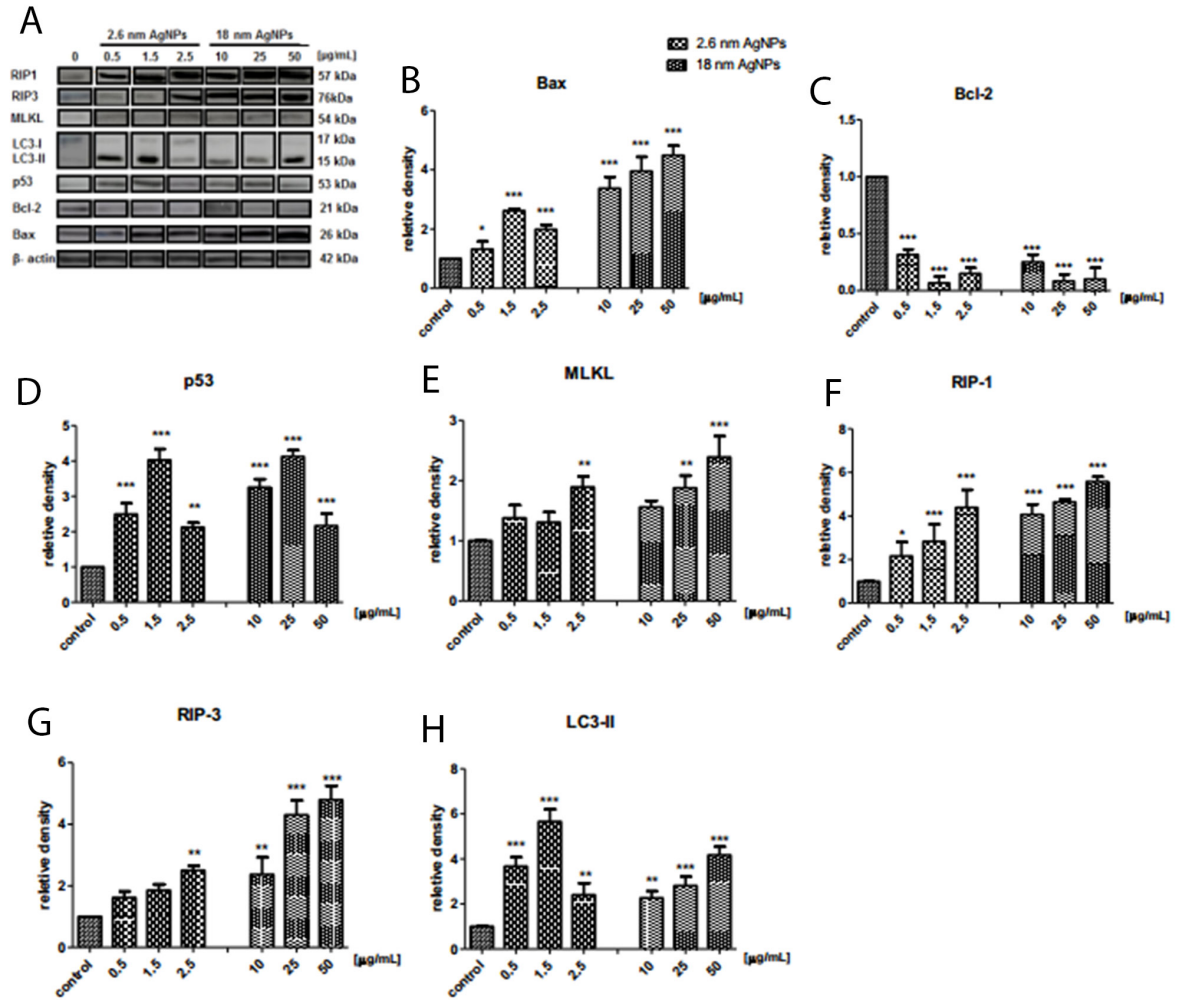
AgNPs-induced changes in protein level of cell death markers in PANC-1 cells Representative Western blot analysis of Bax, Bcl-2, p53, MLKL, RIP-1, RIP-3, LC-3 **(A)**. Quantitative analysis of Bax **(B)**, Bcl-2 **(C)**, p53 **(D)**, MLKL **(E)**, RIP-1 **(F)** RIP-3 **(G)**, LC-3 **(H)**. Data are expressed as means ± SD of 3 independent experiments. ^*^p<0.05; ^**^p<0.01; ^***^p<0.001 exposed cells v/s control.

To clarify the implication of apoptosis, autophagy and necroptosis in AgNPs-induced PANC-1 cells death we determined Bax, Bcl-2, RIP1, RIP3, MLKL, and LC3 at protein level. As shown on Figure 13A, the level of pro-apoptotic protein Bax elevated significantly after exposure to 2.6 nm AgNPs with the highest increase at 1.5 μg/mL and after treatment with 18 nm AgNPs in a concentration-dependent manner. Moreover, larger AgNPs caused stronger changes. Similarly, the content of p53 protein increased significantly after exposure to 2.6 and 18 nm AgNPs, with the highest elevation at concentration of 1.5 and 25 μg/mL, respectively (Figure 13D). Whereas the anti-apoptotic/pro-survival Bcl-2 protein level was very low after treatment with AgNPs and this effect was not dependent on the size and concentration of NPs (Figure 13C). This results together with TEM observation indicated implication of apoptosis in AgNPs-induced PANC-1 cell death.

According to our assumption, PANC-1 cells incubation with AgNPs resulted in a size- and concentration-dependent increase of MLKL, RIP1 and RIP3 protein level, which are directly linked to the process of necroptosis (Figure 13E-13G). Statistically significant increase of RIP3 and MLKL protein level was found after treatment with 2.5 μg/mL 2.6 nm AgNPs and after exposure to 25 μg/mL and 50 μg/mL 18 nm AgNPs.

Moreover, the protein level of autophagy marker - LC3-II increased significantly in PANC-1 cells treated with AgNPs, the highest elevation was induced by 1.5 μg/mL of 2.6 nm nanoparticles. AgNPs with size 18 nm induced autophagy, reflected by LC-3-II content, in a concentration-dependent manner (Figure 13H).

This results together with TEM observation indicated that both 2.6 and 18 nm AgNPs induced apoptotic and neroptotic cell death occurring with autophagy in adenocarcinoma pancreatic cancer cells.

## DISCUSSION

In recent time, the applications of AgNPs have risen up in oncology diagnostics and treatment as a drug delivery system [42, 43]. Nonetheless, AgNPs activity towards cancer cells seems to be still under investigation. In the present study, we have examined the cytotoxic effect of AgNPs towards pancreas ductal adenocarcinoma cells (PANC-1) compared to non-tumor immortalized pancreas duct cells (hTERT-HPNE). The PANC-1 cell line is one of the most studied and well-characterized *in vitro* model of human pancreatic adenocarcinoma [44]. We evaluated AgNPs activity in dependency on their size and concentration focusing on the type of cell death. Performed characterization indicated that AgNPs with both sizes are stable, monodispersed are suitable for study of anticancer potential. We also confirmed that the size of 2.6 and 18 nm was very close to that declared by the manufacturer. Moreover, similarly to observation described by Gliga et al. [45], we detected that smaller AgNPs released more Ag in cell medium after 24 h incubation compared with the bigger ones. However, the amount of Ag released from both 2.6 nm AgNPs and 18nm AgNPs was low and did not exert cytotoxic effect against PANC-1 or hTERT-HPNE cells. This results is in agreement with our previous conclusions [27].

We have found that AgNPs with both sizes reduced the viability of PANC-1 cells and induced PANC-1 cell death. It has been previously observed that AgNPs showed a strong inhibitory effect on the growth of lung tumor cells (H1299), human tongue squamous carcinoma (SCC-25), human breast cancer cells (MCF-7) and chronic myeloid leukemia (K562) cells [23–25, 43]. He et al. [23] demonstrated antitumor activity of 8-22 nm AgNPs against lung cancer H1299, prostate cancer VCaP, and pancreas cancer BxPC-3 cell lines using MTT assay and the IC_50_ value was 5.33±0.37, 87.33±4.80, and 38.9±2.10 μg/mL, respectively. Furthermore, we investigated the effect of Ag^+^ on PANC-1 and hTERT-HPNE cells. Our data showed a similar values of IC_50_ obtained for 2.6 nm AgNPs and Ag^+^. On the other hand, Ag ions were more toxic than 18 nm AgNPs to both pancreatic cells. In our previous study, we demonstrated that AgNO_3_ exerted more cytotoxic effect against human gingival fibroblast cells in comparison to 10 nm AgNPs [47]. Similarly, Foldbjerg et al. demonstrated that Ag^+^ was approximately four times more cytotoxic to human monocytes than 69 nm PVP-coated AgNPs [48]. Also, it has been indicated that Ag^+^ decreased mitochondrial activity in lung cancer cell more than 69 nm PVP-coated AgNPs with about twofold difference in EC_50_ values [49]. Moreover, morphological assessment of apoptotic cells indicated a dose-response effects of AgNPs on inducing apoptosis of H1299 cells. These results were associated with the inhibition of NF-κB activity, decrease in Bcl-2, and caspase-3 expression. The same authors during *in vivo* study showed that AgNPs could effectively inhibit and slow down the growth of lung tumors in xenograft severe combined immunodeficient (SCID) mouse model [23]. Furtermore, Loutfy et al. [25] demonstrated that treatment with AgNPs of 5-10 nm and 13-15 nm inhibited human breast cancer cell (MCF-7) proliferation in a concentration-dependent manner with IC_50_ value of 6.28 μM, and 14.48 μM, respectively. DNA fragmentation, as presented by electrophoresis and flow cytometry, indicated induction of apoptosis in MCF-7 cells after exposure to AgNPs. Urbańska et al. [50] have demonstrated a significant inhibitory effect of 70 nm AgNPs at concentration of 50 and 100 μM on glioblastoma multiforme (U-87) cells proliferation in *in vivo* model. Our study has emphasized a significant difference in AgNPs toxicity to tumor and non-tumor pancreatic cells. Although, selective cytotoxicity is one of the important criteria for a drug in safety antitumor therapy only a few studies directly compared the relative cytotoxicity of AgNPs on cancerous and non-cancerous cells. Swanner et. al. [51] described cytotoxic effect of AgNPs on triple-negative breast cancer cells at concentration that exerted little effect on nontumorigenic breast cells. Guo et al. [46] found that AgNPs may be approximately 2-fold more cytotoxic to acute myeloid leukemia compared to healthy human bone marrow cells. Similarly, we demonstrated by IC_50_ values obtained from measurements of mitochondrial function (MTT assay), cell membrane damage (LDH assay) that PANC-1 cells are more susceptible to cytotoxicity of AgNPs than hTERT cells. This results provides a clear rationale for further investigation on the potential application of AgNPs in pancreatic cancer treatment. Importantly, also in our previous study we demonstrated that both 2.6 and 18 nm AgNPs affected the viability of non-tumor human gingival fibroblast cell and human osteoblast cells at higher concentrations than pancreatic cancer cells [26, 27]. Moreover, in our study we found that AgNPs-induced suppression of cells proliferation is not only concentration- but also size-dependent. Smaller AgNPs (2.6 nm) exhibited stronger cytotoxic effects against pancreatic cells than bigger (18 nm) ones. This result confirmed reports emphasizing the importance of size of nanoparticles in relation to their toxicity [52]. For example, Liu et al. [53] show that among three sizes of AgNPs, the smallest ones (20.6 ± 2.7 nm) had significant effect on enhancing apoptosis rate and thermo-induced killing of glioma cells *in vitro*. But, Kim et al. [15], in contrast to our finding, demonstrated that among 10 nm, 50 nm and 100 nm AgNPs, the biggest ones exerted stronger cytotoxic effect, detected by the leakage of LDH into cell culture medium, on osteoblast cells (MC3T3) and rat pheochromocytoma cells (PC12). As far as we know, 2.6 nm nanoparticles are the smallest that has been studied to determine their usefulness in anticancer therapy. What is more, AgNPs showed more than a hundred thousand-fold higher efficiency compared with gemcitabine to decrease pancreatic cancer cell viability and induced pancreatic cancer cells death. So far, no effective method of treating pancreatic cancer was developed, and chemotherapy, used as adjuvant treatment, does not give the expected results. Accordingly, AgNPs could give a boost be to the treatment of this deadly cancer. The results of cytotoxicity assay showed that cell death associated with metabolic dysfunction in mitochondria and cell membrane disintegration. Such changes may indicate cell death as a result of programmed cell death or necrosis [54, 55].

Moreover, we found that the decrease in pancreatic tumor cell survival was accompanied by inhibition in cellular proliferation. We found a statistically significant effect of AgNPs on DNA synthesis depending on their size and concentration. Similar data were obtain for 20-30 nm AgNPs in breast cancer cells and human liver cancer cells and for 10, 20, 40, 60, 100 nm AgNPs in colon carcinoma cells [51, 56]. AshaRani et al. [57] confirmed direct involvement of AgNPs (6-20 nm) in decrease of DNA replication in glioblastoma cells (U251). Vasanth et al. described anticancer potential of 40 nm AgNPs against HeLa cell type by inhibiting cell replication [58].

Our TEM analysis indicated changes at ultrastructural level in PANC-1 cells in the presence of AgNPs. Both 2.6 and 18 nm AgNPs showed affinity to cellular membranes and, before entering the cell, they were located around the cell membrane. Next, we noticed two ways of AgNPs uptake by PANC-1 cells: micropinocytosis or caveole-mediated endocytosis [59]. Macropinocytosis can be described as membrane-ruffles-mediated endocytosis which in other words is a clathrin-independent endocytosis involving small vesicles [60]. Similarly, Milic et al. [61] have indicated endocytosis as a way for AgNPs penetration into mammalian kidney cells. The same mechanism of uptake nanoparticles into human monocytic cells (THP-1) was described. On the other hand, it has been also found that nanoparticles may enter cells via several different mechanisms, such as phagocytosis [59]. The uptake of AgNPs have been observed in the cytoplasm of PANC-1 cells. As a result they caused changes typical for apoptosis. Interestingly, we also observed round, swelling nucleus - typical for programmed necrosis – necroptosis [62]. Besides, we noticed changes typical for necrotic cell death. All this types of cell death were associated with formation of autophagosomes. Despite the fact that the cytotoxic and anticancer effect of metal nanoparticles, including AgNPs, has been generally proven, there is still a lack of comprehensive information on their molecular mechanisms of action [2, 63]. However, the role of apoptosis in AgNPs-induced glioblastoma multiforme (GBM), lung cancer and osteosarcoma cells death was emphasized [50, 64, 65]. It has been indicated that this effect correlated with AgNPs impact on NF-κB, Bcl-2 and caspase-3 [23, 66]. Laha et al. [67] described apoptosis and autophagy cell death in human breast cancer cells treated with copper oxide nanoparticles (CuONPs). Our results are consistent with presented information and indicated that 2.6 and 18 nm AgNPs-induced apoptosis associated with increased level of pro-apoptotic Bax protein and decreased level of anti-apoptotic Bcl-2 protein in pancreatic cancer cells. Similarly, Dziedzic et al. [24] found that human squamous carcinoma cells exposed to 10 nm AgNPs resulted in an increase of pro-apoptotic proteins (Bax). It is known that ratio of Bax/Bcl-2 proteins plays a major role in mitochondrial outer-membrane permeabilization, release of cytochrome C into the cytosol and, thus, initiation of apoptosis [68]. Moreover, apoptosis dysfunction plays a key role in the development and progression of cancer and the ability of tumor cells to avoid apoptosis is currently one of the explanation why cancer therapies fail. Therefore, induction of apoptosis or other forms of programmed cell death to selectively kill cancer is one of the key objective of chemotherapy today [69, 70]. Recent studies suggest that p53 protein level is critical in the cellular response to apoptosis [70–72]. We noticed that both 2.6 and 18 nm AgNPs caused an elevation of p53 protein level in PANC-1 cells. This result correlated with data presented by Ye et al. [73] in normal human hepatic cell line (L-02) exposed to nano-SiO_2_ colloids and Mroz et al. [74] for 14 nm nanoparticulate carbon black in adenocarcinomic human alveolar basal epithelial cells. Also, AgNPs are known to induce p53-mediated apoptosis in human breast cancer cells [51]. Furthermore, p53 protein triggers a cell cycle arrest providing time either for damage to be repaired or for self-mediated apoptosis [70]. Probably, increased level of p53 protein associated with increased Bax and decreased Bcl-2 level is one of the mechanisms of programmed cell death caused by AgNPs in PANC-1 cells (dependent on the size and concentration). Indeed, tumor-suppressor p53 has been implicated in several modes of cell death, including also necroptosis, autophagic cell death and mitotic catastrophe [75]. It has been ascertained that a p53 target gene encoding a lysosomal protein that activates macroautophagy, as an effector of p53-induced death in cancer cells [76]. On the other hand, Feng et al. indicated that activation of p53 enhanced autophagy levels in cells, which might contribute to the tumor suppressor functions of p53 [77]. Amaravadi et. al. [78] observed that inhibition of autophagy together with p53 activation lead to therapy-induced apoptotic cells death in a Myc-induced model of lymphoma generated from cells derived from p53ERTAM/p53ERTAM mice (resistant to apoptosis after treatment with tamoxifen). We found that autophagy, measured by LC3-II protein level, assisted AgNPs-induced apoptosis in PANC-1 cells. Similar results was described by Zhang et al. [59] in ovarian cancer cells exposed to salinomycin and AgNPs. Also, Naumann et al. [10] found an induction of both autophagy and apoptosis in pancreatic cancer cells after treatment with sulforaphane. On the other hand, an increase in LC3-II level was also indicated when apoptosis has been inhibited or impaired. [79, 80]. It has been found that inhibition of autophagy may be a new therapeutic approach in the treatment of pancreatic cancer [81]. Fujii et al. [82] reported that activated autophagy is associated with pancreatic cancer cells and correlates with poor patient outcome. Interestingly, Yang et al. [83] indicated that autophagy could suppress tumor at initial stage and support tumor growth later. Thus, the roles of autophagy in cancerogenesis seems to be contradicting. However, it has to be noticed that synergism between nanoparticles and autophagy may improve existing cancer therapies [84]. Several studies indicated that elevated autophagy after nanoparticles treatment leads to increased tumor cell death [85]. Li et al. [12], concluded that activation of both autophagy and apoptosis by nanoparticles suggests that nanoparticle-induced autophagy causes irreversible cellular damage. Indeed, in our study we have observed that 2.6 and 18 nm AgNPs-induced apoptotic as well necroptotic PANC-1 cell death was associated with increased level of autophagy markers (LC-3) and formation of autophagosomes. Also a statistically significant enhancement of p53 proteins level was confirmed for all used concentrations. Furthermore, level of protein Bcl-2, which besides apoptosis also inhibits induction of autophagy, decreased in response to treatment with AgNPs at all used concentration. It has been demonstrated that autophagy is deleterious to tumor cells and promoting apoptotic cell death in response to gemcitabine in PANC-1 and MIA PaCa-2 cells [9]. Interestingly, Giovannetti and Giaccone [86] indicated autophagy as an effective way to induced cancer cell death in selected subgroups of patients with pancreatic ductal adenocarcinomas. Moreover, they found that autophagy induction reduced proliferation, migration and invasion of pancreatic ductal adenocarcinoma cells. Also, Sun at al. [87], documented that MIR506 exerted a tumor suppression effect in pancreatic ductal adenocarcinoma by inducing autophagy-related cell death. The pro-death role of autophagy in AgNPs-induced cytotoxicity was described in lung cancer cells and glioma cells [88, 89]. However, autophagy induced by AgNPs in pancreatic tumor cells has not been described yet.

Moreover, our research has shown that usage of necroptosis inhibitor significantly reduce AgNPs cytotoxic effect on PANC-1 cells. Necrostatin-1 blocks RIP1 kinase and inactivates interaction between RIP1 and RIP3 leading to necroptosis inhibition [31]. The mixed lineage kinase domain like protein (MLKL) is important substrate of RIP3, and, as a consequence, actively participates in necroptosis process [90]. Increased levels of MLKL, RIP1, and RIP 3 proteins clearly demonstrated necroptosis implication in AgNPs-induced PANC-1 cell death. Interestingly, we have identified mixed type of pancreatic cancer cells death, including apoptosis, necroptosis and autophagy after treatment with 2.6 and 18 nm AgNPs for 24 h.

Previously, Yan et al. [91] demonstrated induction of multiple types of programmed cell death: autophagy, apoptosis and necroptosis in bladder cancer cells (T24) after exposure to Troglitazone. Huang et al. reported that ZD55-Interferon-β induced both apoptosis and necroptosis in cancer cells [92].

Interestingly, our TEM observation of PANC-1 cells treated with 0.5 μg/mL 2.6 nm AgNPs revealed cells with multinucleation nuclei. This results indicated implication of mitotic catastrophe in cytotoxic activity of AgNPs [19]. Indeed, it has been indicated that mitotic catastrophe appears at relatively low concentrations of anticancer agent (drug) and can activate multiple distinct modes of cell death, i.e., apoptosis, necrosis and necroptosis [19, 93]. Also, Subramaniam et al. [17] suggested that mitotic catastrophe may be correlated with apoptosis in pancreatic cells treated with curcumin. This finding was confirmed by de-Sá-Júnior et al. [94] whose demonstrated that capsaicin analogue induced apoptosis and mitotic catastrophe in MCF-7 breast cancer cells. Furthermore, Jung et al. [95] described the mitotic catastrophe as a mechanism of lung cancer cells (A549) death after exposure to polymeric nanoparticles containing taxanes.

As far as we know this is the first time when studies present data on mix type of cells death induced by nanoparticles in pancreatic cancer cells. This ability of AgNPs may become a new therapeutic approach to defeat a key mechanism of chemoresistance in pancreas ductal adenocarcinoma cells.

## MATERIALS AND METHODS

### Reagents

AgNPs, water dispersion (2 nm and 15 nm, according to the manufacturer; 2.6 and 18 nm according to our characterization, performed as described below) were purchased from US Research Nanomaterials, USA. The concentration and incubation time, used in these experiments were selected according to our previous study [26, 27]. Gemcitabine were obtained from hospital pharmacy as a remnant of chemotherapy (Gemcitabine for Injection by Accord Healthcare Inc. LOT s08647).

### Characterization of AgNPs

Characterization of AgNPs is presented in our previous study [26, 27]. Additionally, the zeta potentials, polydisperity index (PDI) and the hydrodynamic diameter of the 18 nm AgNPs were detected by Dynamic light scattering (DLS) with a Zetasizer Nano ZS (Malvern Instruments, Malvern, UK). Briefly, AgNPs at concentrations of 10, 50 and 100 μg/mL [28] were dispersed in serum-free (SF) culture medium for both PANC-1 and hTERT and the measurements were made within 1 hour and/or after 24 hours incubation with medium at 37°C and 5% CO_2_. Measurements were performed five times at room temperature. However, significant limitations of DLS as a technique to characterize small nanoparticles (<10 nm) has been demonstrated [29–31]. DLS method is not recommended for characterization of NPs with size of 2.6 nm. Therefore, we characterized 2.6 nm AgNPs using transmission electron microscope Tecnai F20 S/TEM FEG 200kV equipped with energy-dispersive X-ray spectroscopy EDS (Philips Electron Optics, Holland). Prior to the TEM examination AgNPs have been dispersed in water (concentration 1 mg/mL) and placed on a formvar coated, meshed copper grids. This method we have also used to characterize 18 nm AgNPs. The mean size and size distribution of 2.6 nm nanoparticles was determined from multiple randomly selected areas of bright-field TEM images, by measuring many individual particle diameters.

The release of silver ions from nanoparticles (2.6 and 18 nm) was investigated using Inductively Coupled Plasma Mass Spectrometry, (ICP-MS) method [32, 33]. The sample suspensions (1 mL) with AgNPs in growing cell culture medium was ultra-sonicated for 5 minutes at 30°C and 5 mL of uniform sample was transferred to Falcone tube. Silver NPs and silver ions trapped by biomolecules of the medium was removed by centrifuging at 25 000 rpm for 20 min, 0°C; three times. Then 0.5 mL of the supernatant was collected, prepared according to procedure described in our previous study [27] and determined by ICP-MS. A NexION™ 300D ICP-MS (PerkinElmer, Shelton, CT) equipped with a micro flow nebulizer, a quartz cyclonic spray chamber was used for all silver ions concentration determination. The method of calibration curve was used. The standard solutions for calibration curve (50-600 μg/L) was prepared using standard silver concentration of 1 g/L. ICP dedicated silver standard solution in relation to SRM from NIST AgNO_3_ in HNO_3_ 2-3%, 1000 mg/l Ag Certipur® Certified Reference Material, Merck was used. The standard solution was diluted with 2% nitric acid (V). Validation of the spectrometer operation correctness and the measurement procedure was confirmed by analysis of the reference material SRM 1640 Trace Elements in Natural Water (NIST, USA). The resulting silver concentration was consistent with the certificate in terms of uncertainty.

The supernatant, collected after centrifugation of medium preconditioned with AgNPs at 3.5, 5 or 50 and 100 μg/mL (the highest concentration used in the present study), was also incubated with PANC-1 and hTERT-HPNE cells for 24 h in order to evaluate potential cytotoxicity of silver released from AgNPs.

### Cells culture

Human pancreas ductal adenocarcinoma cell line PANC-1 and immortalized human pancreas duct epithelial cell line hTERT-HPNE were used for these experiments. Both cell lines were obtained from the American Type Culture Collection (ATCC). PANC-1 cell line was cultured in Dulbecco's Modified Eagle's Medium (ATCC, Cat. No. 30-2002) with a high concentration of glucose (4.5 g/L), supplemented with 10% fetal bovine serum (FBS) and 1% antibiotics (100 U/mL of penicillin and 100 μg/mL of streptomycin) at 37°C in a humidified atmosphere of 5% CO_2_. hTERT-HPNE cell line was cultured in a mixture of Dulbecco's Modified Eagle's Medium without glucose (Sigmaich- Aldrich, Cat. No. D-5030) and Medium M3 Base (Incell Corp, Cat. No. M300F- 500) (3:1 ratio) with 2 mM L-glutamine adjusted to 1.5 g/L sodium bicarbonate and supplemented with 5% FBS, 10 ng/ml human recombinant EGF, 5.5 mM D-glucose (1g/L) and 750 ng/mL puromycin, at 37°C in a humidified atmosphere of 5% CO_2_.

### Treatments

PANC-1 and hTERT-HPNE cells were incubated with 2.6 nm AgNPs at concentration of 0.5 - 5 μg/mL and 18 nm at concentration 5 - 100 μg/mL for 24 hours. AgNPs were diluted in appropriate serum free (SF) culture media before addition to the cells. In order to avoid AgNPs aggregation, according to the supplier's protocol, nanoparticles dispersion were shaken 1 min prior usage. Additionally, to compare cytotoxicity of AgNPs and Ag^+^, we performed MTT assay. PANC-1 and hTERT-HPNE cells were treated with AgNO_3_ (as a source of Ag^+^) at concentration 0.5-10 μg/mL for 24 h.

### Cell viability

PANC-1 and hTERT-HPNE cells were seeded at a density of 1×10^4^ per well in 96-well plates for the MTT (3-(4,5-dimethylthiazol-2-yl)-2,5-diphenyltetrazolium bromide) assay. After 24 h incubation, the supernatant was removed. Cells were treated with AgNPs and AgNO_3_ (for comparison) at concentration indicated in section: *Treatments* (the final volume 100 μl in each well) as well as with Ag released from AgNPs, prepared as indicated in section *Characterization of AgNPs*. Cell were incubated in 5% CO_2_ at 37°C for 24 h. After 24 h incubation, the media was replaced with 10 μL of MTT stock solution (5 mg/mL) and incubated in 5% CO_2_ at 37°C for 4 h. Afterwards, medium was removed and dimethyl sulfoxide (50 μL) was added to each well for 15 min, and the cell viability was determined by measuring the absorbance of the samples at 570 nm using a microplate-reader (reference wavelength: 690 nm). A blank absorbance values, as read from cell-free wells, were subtracted from the absorption values of each test sample. Also, the influence of gemcitabine (as a positive control) at concentration of 5-100 μM on PANC-1 cell viability was assessed. The results were expressed as a percentage of control.

### Cell death

Cell death was measured using LDH (lactate dehydrogenase) assay (CytoTox 96 Assay, Promega, Poland) according to manufacturer's protocol. PANC-1 and hTERT-HPNE cells were seeded at a density of 10^4^ cells/well into 96-well plates. The next day, both of cells lines was treated under SF conditions with 2.6 nm AgNPs and 18 nm AgNPs (at concentration indicated in section: *Treatments)* for 24 hours or with Ag released from AgNPs, prepared as indicated in section *Characterization of AgNPs*. We also determined the influence of gemcitabine (as a positive control) at concentration of 5-100 μM on PANC-1 cells death. For some experiments - necrostatin-1 at concentration 10 μM, a potent and specific necroptosis inhibitor (Sigma-Aldrich, Poland), was added 2 hour prior the addition of AgNPs to the cells. The concentration of necrostatin-1 was selected based on literature data [34, 35] and preliminary data. Preincubation of cells with 10 μM necrostatin-1, in the absence of AgNPs, exerted no significant effects on cell death (data not shown). After that, 50 μl of cell supernatant was transferred to new 96-well plate containing 50 μl of tetrazolium salt reaction and incubated for 30 minutes at room temperature in dark condition. Then 50 μl of stop solution was added to each well and the spectrophotometric absorbance of the colored formazan was measured at 490 nm wavelength. As a control for maximum LDH release (control (+)), cells were treated with lysis buffer (2% triton X-100) for 10 min before running the assay. Untreated cells served as control (-) for spontaneous LDH release. Absorbance values were corrected with blank NPs. AgNPs-induced cell death was expressed as percentage of LDH found in the culture medium with respect to the total LDH according to the following equation:[36]

$$\text{Percent Cytotoxicity}=100\times \frac{\left(\text{Experimental}-\text{Culture Medium Background}\right)}{\left(\text{Maximum LDH Release}-\text{Culture Medium Background}\right)}$$

### Cell proliferation

Proliferation of PANC-1 and hTERT-HPNE cells was determined *in vitro* using a BrdU proliferation ELISA kit (Roche). PANC-1 cells were seeded in 96-well plates at a density of 1 × 10^4^ cells/100 μl per well. After 24 h incubation, the supernatant was removed and AgNPs in SF culture medium at concentration presented in section *Treatment* was added (the final volume 100 μl in each well) and incubated at 37°C for 24 h. After incubation, 10 μl/well BrdU labeling solution was added and the cells were reincubated for additional 3 h at 37°C. Afterwards, labeling medium was removed and FixDenat Solution was added to the cells for 30 min at room temperature. Next, FixDenat solution was removed and anti-BrdU-POD working solution was added for 90 min at room temperature. Then, antibody conjugate was removed and wells were rinsed three times with 200 μl/well Washing Solution. At the end, 100 μl/well Substrate Solution was added for 15 min at room temperature. Reaction was stopped by adding 25 μl 1M H_2_SO_4_ to each well. The absorbance of the samples was measured in microplate reader at 450 nm (reference wavelength: 690 nm). The data were expressed as the percentage of untreated cells (control), which was set to 100%. Absorbance values were corrected with blank NPs.

### Ultrastructural studies

Transmission electron microscopy (TEM) has been performed in order to investigate AgNPs cellular uptake and ultrastructural changes in PANC-1 cells. PANC-1 cells were cultured in T-10 cm^2^ plates in appropriate, complete medium until confirmed to be 80 - 90% confluent. Subsequently, cells were treated with 2.6 or 18 nm AgNPs at concentration 0.5, 1.5, 2.5, 3.5 μg/mL or 15, 25, 50 μg/mL, respectively for 24 hours and after this step cells were fixed in 2.5% glutaraldehyde in 0.1 mM sodium-cacodylate buffer. Next, cells were detached by scraping, centrifuged and the cell pellets were postfixated in 2% osmium tetroxide and dehydrated in graded series of ethanol. After infiltration with propylene dioxide: epon mixture and pure epon cells were embedded to polymerize. Finally, the ultra-thin sections (Reichert OmU3 ultramicrotome, Austria) were contrasted using uranyl acetate and lead citrate prior to examination in transmission electron microscope at 100kV (JEM 1200EX II, Jeol, Japan).

### Apoptosis detection

Apoptotic and necrotic cells were detected by Annexin V binding and propidium iodide (PI) uptake using apoptosis assay kit (BD Pharmingen, USA). PANC-1 cells were seeded on a 6-well plate at a density of 5×10^6^ cells/well. The next day cells were treated with 2.6 nm or 18 nm AgNPs at concentrations: 0.5, 1.5, 2.5, 3.5, 5 μg/mL or 5, 10, 25, 50, 100 μg/mL for 24 h. After that, cells were collected, washed twice with PBS (NaCl 0.138 M; KCl 0.0027 M; pH 7.4) and resuspended in binding buffer (50 mM HEPES: 4-(2-hydroxyethyl)-1-piperazineethanesulfonic acid, 700 mM NaCl, 12.5 mM CaCl_2_, pH 7.4). 5 μL of Annexin V and 5 μL propidium iodine were added to the cells and gently shaken. After 15 minutes incubation at the room temperature in dark, cells were diluted in the binding buffer and analyzed using a BD FACSArray (BD Biosciences, San Jose, CA, USA). Twenty thousand specific events were analyzed by a fluorescence activated cell sorter analysis in a flow cytometer. A plots from the gated cells illustrated the populations of viable (Annexin V-PI-) cells, apoptotic (Annexin V+PI-), apoptotic/necroptotic (Annexin V+ PI+) cells and dead (Annexin V- PI+) cells.

### Western blotting analysis

Western blotting method was used in order to study protein levels of Bax, Bcl-2, RIP-1, RIP-3, MLKL, LC3 and p53 protein. PANC-1 cells were cultured in 10 cm Petri dishes and treated 2.6 nm or 18 nm AgNPs at concentration: 0.5, 1.5, 2.5 μg/mL or 10, 25, 50 μg/mL as described in section *Treatment*. After 24 h of incubation, culture media was removed, cells were washed three times with cold PBS (NaCl 0.138 M; KCl 0.0027 M; pH 7.4), collected and centrifuge at 1500 rpm/min for 5 min at room temperature. Then, supernatant was removed, cells were lysed with protein lysis buffer (50 mM Tris pH 7.5, 150 mM NaCl, 1% Triton X-100, 0,1% SDS) in the presence of protease (Roche, Cat. No. 04693159001). Subsequently, cells homogenates were centrifuged at 16 000 rpm/min for 20 min at 4°C. Cell lysate supernatants were collected and stored at -80°C until further use. The protein concentrations in cell lysates were measured by Bradford method [34]. The samples (40 μg protein per lane) were boiled for 5 minutes and separated by SDS–PAGE on polyacrylamide gel and transferred onto nitrocellulose membrane, which was blocked with 5% non-fat dry milk-PBST buffer (phosphate-buffered saline (PBS) containing 0.1% Tween-20) for 1 hour at room temperature and incubated at 4°C overnight with rabbit polyclonal antibody: anti-Bax, anti-Bcl-2, anti-RIP-1, anti-RIP-3, anti-LC3, anti-p53, and goat polyclonal anti-MLKL (diluted 1:500). After serial washes in TBST buffer (Tris-buffered saline with Tween: 137mM NaCl, 2,7 mM KCl, 19 mM Tris base) membrane was incubated at room temperature for 1 h with anti-rabbit or anti-goat secondary antibody (1:20 000). All antibodies used in this study were purchased from Santa Cruz Biotechnology (Santa Cruz, CA, USA), only LC3 antibodies were purchased from Thermo Fisher Scientific (Thermo Fisher Scientific, Uppsala, Sweden). The immunoactive proteins were determined using an enhanced chemiluminescence (ECL) Western blotting detection kit (Amersham Biosciences, Piscataway, NJ, USA). The same membrane was stripped and β-actin (Sigma Aldrich) was used as an internal control. Proteins level were quantified using densitometry software (ImageQuant Software).

### Protein content

The method of Bradford was used to measure protein level [37]. 2 μL of protein sample was added to 1 mL of Bradford reagent. Subsequently, the samples were gently mixed and incubated at room temperature for 20 min. After incubation, the absorbance of the samples at λ = 595 nm was measured with a UV-Vis spectrophotometer (Hach DR3900). The protein content of the sample was determined on the basis of the calibration curve.

### Statistical analysis

The results were expressed as mean ± standard error of 3 independent experiments, and the significance of the difference between the mean values relative to control was determined by the one-way analysis of variance and Tukey's post-hoc test. Significance was determined at the 5% level (^*^ *p* < 0.05), two-sided. Statistical significance between treatment and control group was indicated by asterisk. The analysis of variance (one-way ANOVA) was performed using the procedure provided GraphPad Prism 5 software (GraphPad Software, Inc, La Jolla, CA, USA). The inhibitory concentration (IC_50_) was calculated from the following equation: log(inhibitor) vs responses curve, with an equation: Y = bottom + (top − bottom)/(1 + 10^(Log IC50−X)^ × Hill slope) using the GraphPad Prism 5 program.

## CONCLUSIONS

To summarize, we observed higher cytotoxic effect of 2.6 and 18 nm AgNPs on human pancreas ductal adenocarcinoma cells compared with non-tumor cells of the same tissue. PANC-1 cell death was associated with morphological changes and biological events. Our finding documented that treatment with AgNPs resulted in a significant, concentration-dependent inhibition of pancreatic cancer cell proliferation as well as induction of apoptosis and necroptosis. Furthermore, we observed cellular uptake of AgNPs and ultrastructural changes in PANC-1 cells, typical for apoptosis, necroptosis/necrosis as well as autophagic and mitotic catastrophe. These alterations were associated with a significant increase in the level of autophagy (LC3-II), necroptosis (RIP1, RIP3, MLKL) and apoptosis (Bax) proteins as well as decreased level of anti-apoptotic marker (Bcl-2). Additionally, we showed elevated level of tumor suppressor p53 protein, characteristic for apoptosis, autophagy and necroptosis. In conclusion, we identified programmed cell death: apoptosis and necroptosis, associated with autophagy and mitotic catastrophe induced in PANC-1 cells by AgNPs in a concentration- and size-dependent manner. Our results lead us to assume that AgNPs could bypass drug resistance by inducing mixed type of cell death in pancreatic ductal adenocarcinoma cells.

## SUPPLEMENTARY MATERIALS FIGURES


