# Supplementary material for: Silver nanoparticles of different sizes induce a mixed type of programmed cell death in human pancreatic ductal adenocarcinoma
Source: Oncotarget. 2017 Nov 20;9(4):4675–97. doi: 10.18632/oncotarget.22563 (PMC5797005; doi:10.18632/oncotarget.22563)
Supplement: Supplementary file 1 [file oncotarget-09-4675-s001.pdf]

Silver nanoparticles of different sizes induce a mixed type of programmed cell death in human pancreatic ductal adenocarcinoma

SUPPLEMENTARY MATERIALS

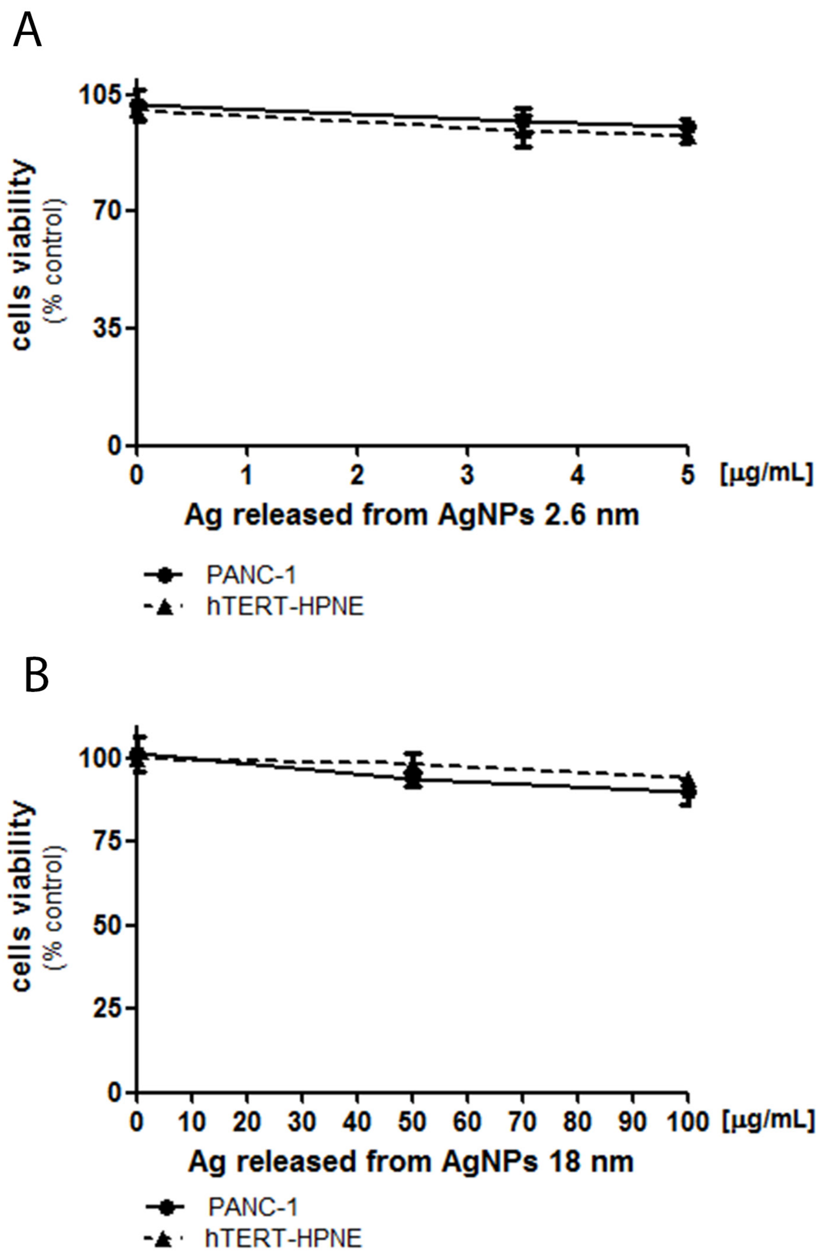

Supplementary Figure 1: Ag released in cell culture medium from AgNPs did not affect PANC-1 and hTERT-HPNE cells viability. Ag released in cell culture medium from a 2.6 nm AgNPs at concentration of 3.5 or 5 µg/mL and b 18 nm AgNPs at concentration of 50 or 100 µg/mL after 24 h of incubation did not affect PANC-1 and hTERT-HPNE cells viability. Data are expressed as means ± SD of 3 independent experiments.

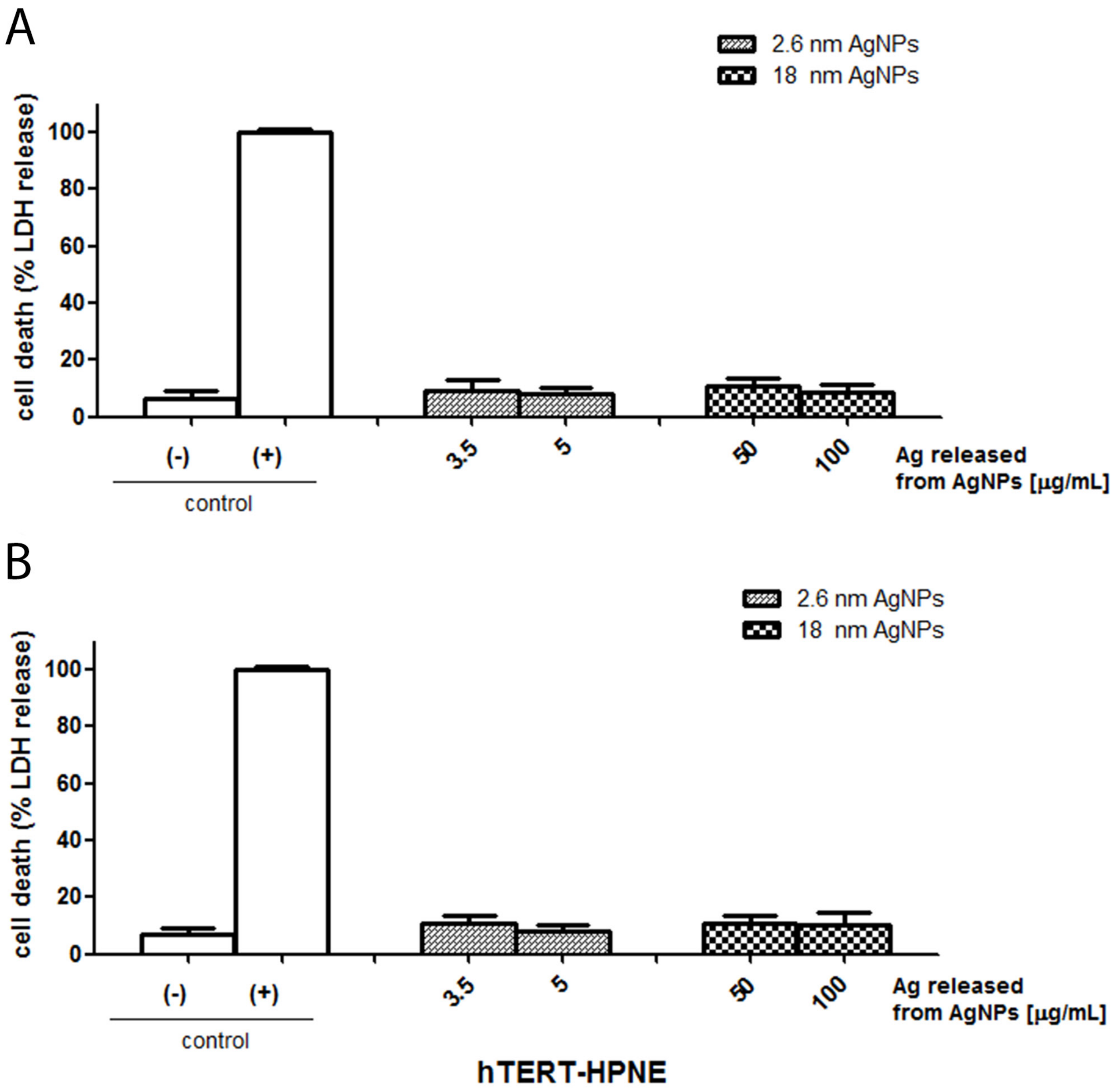

Supplementary Figure 2: Ag released in cell culture medium from AgNPs did not affect PANC-1 and hTERT-HPNE cells death. Ag released in cell culture medium from a 2.6 nm AgNPs at concentration of 3.5 or 5 µg/mL and b 18 nm AgNPs at concentration of 50 or 100 µg/mL after 24 h of incubation did not affect PANC-1 and hTERT-HPNE cells death. As a control for maximum LDH release cells were treated with lysis buffer (control (+)); untreated cells served as control (-) for spontaneous LDH release. Data are expressed as means ± SD of 3 independent experiments.

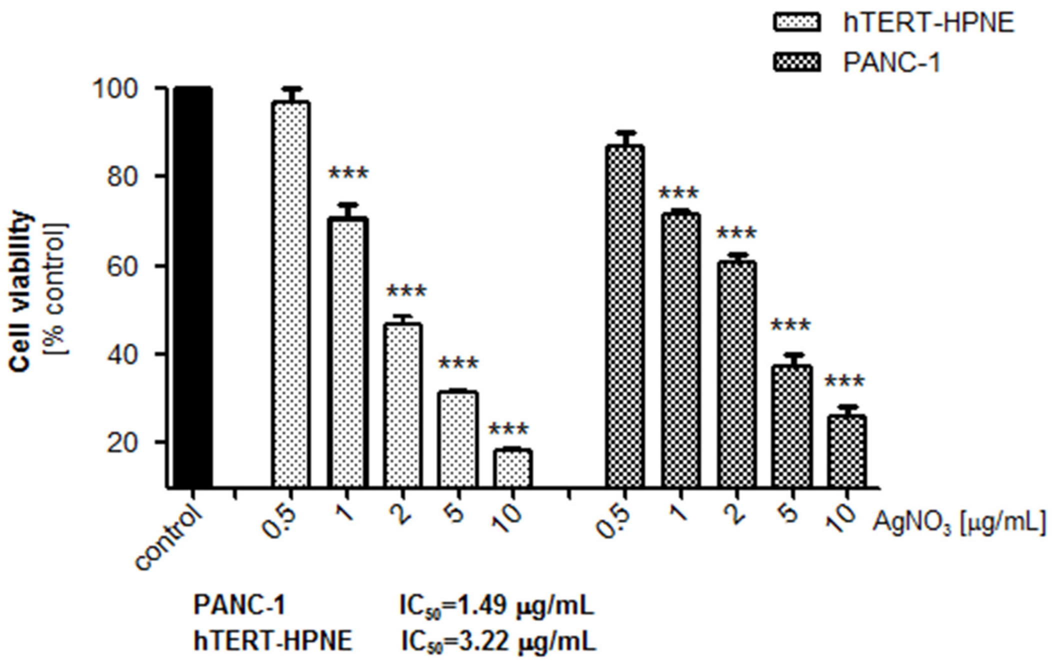

Supplementary Figure 3: AgNO3 (used as a source of Ag+) decreased PANC-1 and hTERT-HPNE cells viability. AgNO3 decreased PANC-1 and hTERT-HPNE cells viability at concentration of 0.5, 1, 2, 5, 10 µg/mL after 24 h of incubation. Data are expressed as means ± SD of 3 independent experiments; ***p<0.001. The inhibitory concentration (IC50) was calculated from the following equation: log(inhibitor) vs responses curve, with an equation: Y = bottom + (top − bottom)/(1 + 10(Log IC50−X) × Hill slope) using the GraphPad Prism 5 program.
